# Texture Preferences of Chinese, Korean and US Consumers: A Case Study with Apple and Pear Dried Fruits

**DOI:** 10.3390/foods9030377

**Published:** 2020-03-24

**Authors:** Runrou Wong, Seulgi Kim, Seo-Jin Chung, Mi-Sook Cho

**Affiliations:** Department of Nutritional Science & Food Management, Ewha Womans University, 52, Ewhayeodae-gil, Seodaemun-Gu, Seoul 03760, Korea; nikowong0222@gmail.com (R.W.); seulgikim@ewhain.net (S.K.); misocho@ewha.ac.kr (M.-S.C.)

**Keywords:** fruit chips, hedonic based projective mapping, hedonic transfer, cross-culture, consumer liking

## Abstract

The present study aimed to understand the drivers of liking dried apple and pear chips with various textures among Chinese (*n* = 58), Korean (*n* = 58), and US (*n* = 56) consumers. The possibility of hedonic transfer from snack texture preferences to fruit-chip texture preferences was also investigated among Chinese and Koreans. Fourteen fruit-chip samples with four textural properties (crispy, puffy, soft, and jelly-like) were selected. Consumers rated their level of liking for each sample, and then they performed hedonic-based projective mapping with the same samples. In the hedonic texture transfer investigation, consumers rated their acceptance of nine snacks with various textures but possessing similar textures to those of dried fruit samples. The data were analyzed by ANOVA and multiple factor analysis. Most consumers disliked samples with a soft or jelly-like texture, while liked samples with a crispy texture. Cross-cultural differences were observed in the liking of puffy samples, with both Chinese and Koreans liking puffy samples as much as crispy ones for their melting characteristics in the mouth, while US consumers perceived the puffy samples as being Styrofoam-like and disliked them. Hedonic transfer was observed from snack texture preferences to fruit-chip. Individual texture preferences for snacks seem to significantly affect the texture preferences for fruit chips.

## 1. Introduction

One of the problems facing Korean agricultural businesses is the steady decrease in the consumption of fresh domestic fruits, including apples and pears, which is partly due to increases in the amount of fruit being imported [1]. Specifically, imported bananas and oranges are replacing the consumption of domestic apples and pears [1,2], and so the fruit industry in Korea is actively seeking strategies to increase the consumption of these traditionally grown domestic fruits. Value-added fruit products are a very attractive marketing concept since they meet increasing consumer demands for healthy and natural products [3,4,5]. Efforts are being made to develop novel fruit products utilizing innovative processing techniques [6,7,8,9,10]. Dried fruit chips have recently become one of the most prominent product categories due to them being marketed as convenient and healthy snack alternatives [11], and the compound annual growth rate of fruit snacks is anticipated to exceed 8% between 2019 and 2025 [12]. Mainland China and US are especially attractive markets for Korea since these two countries are two of the top three countries importing Korean food products [13].

Drying not only extends the shelf life of fresh fruit but also concentrates health functional substances such as antioxidants [14]. Moreover, the sensory characteristics of dried fruit products can be altered markedly even within the same fruit type by using different drying methods [15]. This could create numerous opportunities for fruit industries to develop a broad spectrum of products with various flavor and texture properties. The quality parameters for fresh fruits such as pear and apple are relatively straight forward. Optimal sugar to acid ratio, juiciness, and firmness are some of the key attributes influencing consumer acceptance positively [16,17]. On the contrary, the drivers of liking for dried fruit chips can be less predictable since various textures as well as flavors can be created through different processing methods mentioned above.

Texture is an extremely important sensory modality influencing the liking of a solid food product. Attributes such as crispiness, crunchiness, and viscosity have been reported as key factors affecting the liking of snacks, fruits, and dairy products [18]. Zou et al. [19] reported that a pleasant crispy mouthfeel was one of the reasons for the increased popularity of fruit chips among consumers. Although many researchers adopted cross-cultural designs to understand consumer’s liking for various foods, most previous studies have focused on flavor rather than texture aspects due to the native characteristics of target food products [10,20,21,22,23].

Various hypotheses have been proposed for the reasons underlying consumer preferences for certain foods. For example, the relationships between consumer genetic sensitivities to specific tastants and food liking have been extensively studied [24,25]. Familiarity with a certain sensory quality is frequently reported to be a prominent factor for explaining food preferences [26,27,28]. Familiarity (or previous experience) for a food product, flavor, or texture has been a useful component for delineating the cross-cultural discrepancies in food acceptances. Cross-cultural differences in liking are often observed when the familiarity for the target food items differs among different countries. In contrast, cross-cultural agreement in liking are reported when the target food (i.e., fresh apple and apple juice) item is consumed commonly in different countries [16,29].

Additionally, it has been demonstrated that liking a specific taste can serve as an effective predictor for the acceptance of a foodstuff that exhibits the corresponding taste at a high level [30]. For example, likers of sweetness were shown to give higher acceptance scores for sweet foodstuffs with higher sucrose concentrations. In the present study we defined this phenomenon as hedonic transfer, with consumer preferences for certain modalities (e.g., sweetness or bitterness) not being restricted to a specific food category, but instead transferring to other product categories. It may be worth investigating whether such hedonic transfer can also be observed in the context of texture, where the preference for a specific texture in snacks can serve as a predictor for fruit-chip texture preferences.

General projective mapping (also called Napping) [31,32] and variants of this method have been used widely to understand the perceptual configuration of consumers toward target products based on the similarities and dissimilarities [33] when there is a relatively large number of products to investigate. Projective mapping combined with ultraflash profiling (UFP) has become a widely accepted method for profiling the sensory characteristics of target products from the perspective of consumers. Projective mapping using a hedonic framework was recently introduced by several researchers and shown to be effective in identifying the drivers of liking for certain product categories [34,35].

The present study investigated the acceptance as well as the perceptual configuration of dried apple and pear chips with various texture properties among Chinese, Korean, and US consumers, with the aim of understanding the key attributes that drive the liking of fruit chips in a cross-cultural context. Additionally, the possibility of hedonic transfer from snack texture preferences to fruit-chip texture preferences was studied among Chinese and Korean consumers. We hypothesized that the consumers with different cultural background will differ in the acceptance of and the drivers of liking for fruit chips. Additionally, the occurrence of hedonic transfer from snack texture to fruit chip texture was hypothesized.

## 2. Materials and Methods

### 2.1. Experimental Overview

This study performed two subexperiments. The first part of the study (Experiment 1) investigated the cross-cultural liking of dried fruits among consumers from China, South Korea, and the US. Consumers were asked to evaluate two types of fruit (apple and pear) produced as fruit chips with four textural properties (crispy, jelly-like, puffy, and soft) in terms of liking and the reasons for liking and disliking by means of hedonic projective mapping. Fourteen samples were evaluated by the Chinese and Korean consumers while the US consumers evaluated 13 samples (1 sample was not available). Thus, 13 products commonly tasted by all consumers were cross-culturally compared for their liking and perceptual configuration.

The second part of the study (Experiment 2) investigated whether the preference for a certain texture in one product category (snack texture preference) would transfer to the texture preference in another product category (dried fruit). Nine types of snack samples with various texture characteristics but sharing texture properties similar to those of the dried fruit samples (i.e., crispy, jelly-like, and puffy) were selected. The same Chinese and Korean consumers who participated in Experiment 1 were asked to evaluate their acceptance of the nine snack samples. Based on the texture preferences for snacks, consumers were grouped into likers of a crispy, jelly-like, or puffy texture. The significance of the relationships between their snack texture preferences and their liking of fruit-chip textures were then analyzed.

This study was approved by the Institutional Review Board (IRB) at Ewha Womans University, Seoul, South Korea (IRB No.: 164-29). The hypotheses of the experiments and analytic plans were specified prior to the actual data collection and statistical analysis.

### 2.2. Consumers

This study used online community bulletin boards to recruit 172 consumers from Korea, China, and the US who were interested in participating in consumer taste tests of dried fruits. Fifty-eight Korean consumers (8 males and 50 females) and 58 Chinese consumers (7 males and 51 females) ranging in age from 21 and 37 years who resided in Seoul, South Korea were recruited. The taste testing experiments were performed in Seoul. Fifty-five US consumers (12 males and 43 females) aged between 18 and 37 years were recruited in the community of Corvallis, OR, USA, where the taste testing experiments were conducted. All participants from Korea and US were citizens of South Korea and USA, respectively. Chinese participants consisted of subjects from Mainland China (81.1%), Taiwan (15.5%), and Hong Kong (3.4%). The mean ages of the Chinese, Korean, and US consumers were 24.7 (STD ± 2.7), 25.1 (±3.1), and 24.2 (±3.7) years, respectively. After completing the taste testing, all consumers received a small token of appreciation for their participation.

### 2.3. Samples

#### 2.3.1. Experiment 1: Cross-Cultural Drivers of Liking for Dried Fruit Chips

Fourteen types of fruit chips (seven pear and seven apple chips) with four textural properties (crispy, jelly-like, puffy, and soft) were selected as the products of interests in the hedonic mapping experiments. All of the dried fruit chips from Korea, China, and the US were purchased online. The textural properties of the dried fruit products were affected by the drying method applied in their production: the crispy dried fruits were either baked or fried after air drying, jelly-like dried fruits were dried using wind or forced air, puffy dried fruits were made using freeze-drying, and soft dried fruits were made by combining air drying and microwaving. Chinese and Korean consumers evaluated all 14 samples, while the US consumers evaluated 13 samples since sample Crisp_P_SNC was not available for the experiments performed in the US (Table 1). Detailed information about the samples is provided in Table 1.

In order to maintain the freshness of the produced fruit chips, the samples were opened 30 min prior to the testing sessions. All samples were cut into bite size (3 × 3 × 0.1~0.3 cm), and 2~5 g of each sample was served. The cut dried fruit chips were put in white disposable plastic cups (diameter of 7.0 cm and height of 4 cm; Samboopack Corporation, Incheon, Korea) and lidded. The samples were coded with 3-digit random numbers and were served in room temperature. The serving orders of the samples were determined by a Williams Latin square design [36]. Bottled spring water and unsalted crackers (Carr’s Original Table Water, United Biscuits, Carlisle, UK) were provided to the consumers for cleansing the palate between sample evaluations.

#### 2.3.2. Experiment 2: Hedonic Transfer of Texture Preference

All 14 types of dried fruit-chip samples in Experiment 1 and 9 types of snacks were selected as the products of interest. Nine types of snacks with various textural properties (i.e., crispy, jelly-like, and puffy) corresponding to those of the dried fruit-chip samples were selected and purchased online (Table 2). Additionally, sweet/sour-flavored rather than savory snacks were chosen for the experiment in order to minimize the influence of the flavor factor when investigating the relationships between the general preferences of the consumers for snack and fruit-chip textures. The sample preparation method, serving size, serving order, serving methods, and evaluation protocol for the snacks were identical to those for the fruit-chip samples.

### 2.4. Procedure

#### 2.4.1. Experiment 1: Cross-Cultural Drivers of Liking for Dried Fruit Chips

The consumers from the three countries evaluated the fruit-chip samples using projective mapping with a hedonic framework [31] in order to compare the perceptual configurations of sample liking and reasons for liking/disliking. The projective mapping evaluation consisted of three sessions: (1) a projective mapping learning session, (2) sample evaluation concerning the liking level and reasons for liking/disliking, and (3) the main projective mapping task with fruit chips using a hedonic framework.

As recommended in previous studies [37], a learning session for the overall projective mapping procedure was held to help consumers understand the evaluation method prior to participating in the main experiment. Consumers practiced performing projective mapping with a hedonic framework using photographs of 10 different flowers during the learning session. In the second session, consumers tasted and rated the liking (overall, appearance, flavor, and texture) of 14 dried fruit-chip samples (13 samples for the US consumers) on a 9-point hedonic scale whose anchor words were ‘dislike extremely’ to ‘like extremely.’ Consumers were also asked to freely write their reasons for liking and disliking each sample. During the third session, consumers performed projective mapping with a hedonic framework on a sheet of paper (40 × 60 cm). That is, they positioned the 14 samples (13 samples for the US consumers) based on the similarities of overall liking levels and reasons for liking or disliking as evaluated during the previous session. Consumers then tasted the samples again to remember and confirm their evaluation from the second session. This procedure resulted in samples being located closer to each other if their liking levels and reasons for liking or disliking were similar, and vice versa. After placing all of the samples on the sheet of paper, consumers were asked to replace the samples with the corresponding three-digit coded stickers and write down the reasons for liking and disliking directly beside each sample on the sheet.

#### 2.4.2. Experiment 2: Hedonic Transfer of Texture Preference

The consumers from Korea and China who participated in Experiment 1 were also asked to taste and rate the nine types of snack samples on the same 9-point hedonic scale on another day in terms of their overall liking, appearance liking, taste/flavor liking, texture liking, and familiarity.

### 2.5. Demographic Information

The consumers filled in a questionnaire on demographic information after completing all of the tests. The questionnaire queried their age, gender, nationality, and general liking and frequency of consumption of fresh fruit, dried fruit chips, and snacks. In the case of measuring the general liking of these products, consumers were asked to list top 3 favorite products in rank order for each of fresh fruit, fruit chip, and snack category.

### 2.6. Statistical Analysis

#### 2.6.1. Experiment 1: Cross-Cultural Drivers of Liking for Dried Fruit Chips

Statistical analysis was carried out with data obtained from 13 samples commonly evaluated by all consumers in the three countries. Analysis of variance (ANOVA) using a general linear model (GLM) was used to determine the effects of sample, consumer nationality, and the interaction between nationality and sample on the scores for overall liking, appearance liking, taste/flavor liking, and texture liking. The following GLM was applied: fruit chip acceptance = dried fruit texture type + nationality + dried fruit texture type × nationality + nationality × panelist. Duncan’s multiple-range test was applied as a post-hoc test when a sample was significant for a specific sensory attribute. The data were analyzed using IBM SPSS Statistics software (version 21, SPSS, Chicago, IL, USA).

Multiple factor analysis (MFA) was used to visually summarize the perceptual sample configuration of the consumers from the three countries. The descriptive terms generated by UFP to express the reasons for liking and disliking each sample were analyzed as supplementary variables in the MFA. For the hedonic projective mapping data, the position of stickers on the sheet of paper were measured as X and Y coordinates relative to the bottom-left corner of the sheet, which were entered as X and Y values in a contingency table. Text mining was used to analyze UFP data by calculating the number of occurrences of each term mentioned by the consumers to describe the reasons for liking and disliking each sample. Comments with long sentences were first shortened to words (e.g., “it is sweet” was simplified to “sweet”) and terms with similar meanings were combined (e.g., “skin or peel” was shortened to “skin”). During this preliminary process, two native speakers of each of Chinese, Korean, and English reviewed, discussed and transcoded the descriptors. After refining the terms for statistical analysis, terms that were mentioned by more than 10% of the consumers in each country were chosen for further analysis. A cut off value of 10% was used to disclose the drivers of (dis) liking since many attributes commented for (dis)liking reasons showed frequencies between 10%~20%. Korean and Chinese descriptors were translated into English, and the finalized descriptors were used as the supplementary variables in MFA. This analysis was performed with the FactoMineR package in R Studio software [38].

#### 2.6.2. Experiment 2: Hedonic Transfer of Texture Preference

As in Experiment 1, ANOVA using a GLM was applied to the data obtained from the consumer acceptance tests of snacks and dried fruits. In the case of dried fruits, all 14 samples were subjected for analysis since Chinese and Koreans evaluated these samples. To find the correlation between snack and dried fruit texture preferences, the snack texture preference of each consumer was identified (i.e., crispy, jelly-like, or puffy texture) based on the highest mean texture liking scores among crispy, jelly-like, and puffy snacks. The data from consumers with no particular snack texture preference were removed prior to the analysis. For example, the data were removed if the consumer’s mean texture liking scores of crispy and puffy snacks were identical and scored the highest. Based on this criterion, data from five Chinese and two Korean consumers were removed. ANOVA using a GLM was then conducted with the following model: fruit chip texture acceptance = snack texture preference + dried fruit texture type + nationality + dried fruit texture type × nationality + snack texture preference × nationality + snack texture preference × dried fruit texture type + snack texture preference × dried fruit texture type × nationality + snack texture preference × nationality × panelist. IBM SPSS Statistics software (version 21, SPSS) was again used to analyze these data.

## 3. Results

### 3.1. Consumption Frequencies of Fresh Fruits, Dried Fruits and Snacks

**Figure 1:**
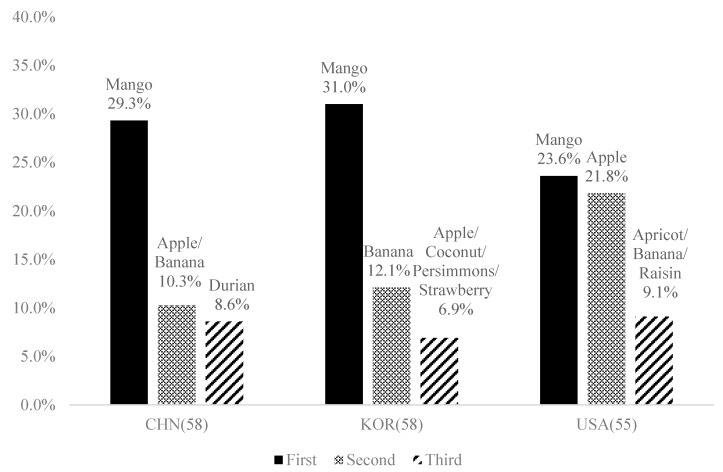
Top ranked dried fruits preferred by Chinese, Korean, and US consumers.

The favorite dried fruit (Figure 1) of the Chinese consumers was mango (29.3%), followed by apple and banana (10.3%) and durian (8.6%). The Korean consumers mostly preferred mango (31%), followed by banana (12.1%) and apple/coconut/persimmons/strawberry (6.9%). Similar to the Chinese consumers, US consumers mostly preferred dried mango (23.6%) and apple (21.8%), followed by apricot/banana/raisin (9.1%). The consumption frequencies of dried fruit were slightly higher for US consumers than for Chinese and Korean consumers. Dried fruits were eaten at least monthly by approximately 70%, 55%, and 35% of the US, Chinese, and Korean consumers, respectively.

The top-three snacks among Chinese consumers were chips (39.7%), extruded snacks (12.1%), and chocolate (8.6%), while those were extruded snacks (39.7%), chips (36.2%), and chocolate snacks (6.9%) among Korean consumers. Unlike the Chinese and Korean consumers, the US consumers liked crackers (27.3%) the most, followed by chips (20%) and a dried fruit snack mixture.

### 3.2. Cross-Cultural Drivers of Liking for Dried Fruit Chips

#### 3.2.1. Cross-Cultural Comparisons of Consumer Acceptance of Dried Fruit Chips

A GLM was used to analyze the effects of consumer nationality, sample, and the interaction between nationality and sample on the acceptance scores (i.e., overall, appearance, taste/flavor, and texture) (Table 3). US consumers generally gave significantly higher scores for the attributes compared to Chinese and Koreans (mean overall liking scores [OLs] of 5.2^a^, 5.4^a^, and 5.7^b^ (values sharing a same alphabet are not significantly different among countries) for Chinese, Korean, and US consumers, respectively); the main exception was for texture liking (OL = 5.1^a^, 5.4^b^, and 5.4^b^, respectively). The nationality and sample interaction effect differed significantly in all of the attributes. Overall, crispy type fruit chips made from apple were commonly scored high in overall liking among consumers in all three countries (Table 4). Nevertheless, cross-cultural differences were observed for the specific acceptance levels of several samples; for example, sample Crisp_A_SNC was liked significantly more by Korean and US consumers than by Chinese consumers, while sample Soft_A_STSR was liked by US consumers but disliked by Chinese and Korean consumers.

Specifically, in terms of overall liking, the Chinese consumers liked samples Crisp_A_SF and Puff_A_AR significantly more than the other samples, but disliked all of the soft dried fruit samples regardless of their fruit type (OL = 4.3, 4.1, and 4.5 for samples Soft_A_IBS, Soft_A_STSR, and Soft_P_STBS, respectively) and also disliked sample Jelly_P_YCNF (OL = 4.4). Korean consumers liked crispy apple samples (OL = 6.3 and 6.2 for samples Crisp_A_SNC and Crisp_A_SF, respectively), all of the puffy apple and pear samples (OL = 6.0, 6.1, 6.1, and 6.2 for samples Puff_A_AR, Puff_A_ICBY, Puff_P_HS, and Puff_P_ICBY, respectively), and sample Jelly_P_GGM (OL = 5.8). All of the other samples were rated below 5.0, with scores clustered between 4.0 and 4.8. US consumers noticeably liked sample Crispy_A_SF (OL = 7.6) among all of the samples. The scores were relatively low for sample Puff_A_ICBY (OL = 5.4), soft dried fruit samples (OL = 4.9 and 4.8 for samples Soft_A_IBS and Soft_P_STBS, respectively), and sample Jell_P_YCNF (OL = 4.7).

A GLM was separately applied to each of the pear and apple chip data sets to investigate whether the consumer liking of chips was affected by their texture type. The results (Appendix A) showed that texture type significantly affected (*p* < 0.05) all attributes for both apple and pear chips in all three countries. Overall, US consumers gave clearly higher mean liking scores for crispy than other texture types for apple and pear chips for most of the attributes (Table 4). In contrast, the Chinese and Korean consumers commonly gave higher liking scores for puffy than for other types of dried fruits. In addition to puffy, the Chinese consumers also liked crispy samples.

#### 3.2.2. Multiple Factor Analysis of the Consumer Perceptual Configuration of Dried Fruit Chips

##### Liking and Disliking Terms Generated for Profiling Dried Fruit Samples 

The Chinese, Korean, and US consumers used 293, 240, and 241 terms, respectively, to describe their reasons for liking the samples. These terms were merged into, 85, 98, and 80 terms, respectively, for the initial analysis. In the case of disliking terms, Chinese, Korean, and US consumers initially used 345, 352, and 380 terms, respectively, which were reduced to 84, 81, and 89 terms. As mentioned above, the combined terms commonly chosen by more than 10% of the consumers were further selected as supplementary variables in the MFA. Thus, 37 terms (19 liking and 18 disliking) for Chinese consumers, 41 terms (18 liking and 23 disliking) for Korean consumers, and 31 terms (13 liking and 18 disliking) for US consumers were used in the final MFA (Table 5). Concerning the terminology used, consumers from all three countries frequently answered “none” as the reasons for liking when the sample was disliked, and also “none” as the reasons for disliking when the sample was liked. The usage frequencies of this term were strongly correlated with the levels of liking and disliking the samples.

##### Cross-Cultural Comparison of Consumer’s Perceptual Configuration of Dried Fruit Chips 

Overall, the MFA map of all three countries defined factor 1 (F1) axis by the degree of liking the samples (higher rated samples on the positive and lower rated samples on the negative axis, respectively) and factor 2 (F2) axis by the texture type of the samples. MFA roughly clustered the samples into three groups: crispy, puffy, and soft/jelly.

**Figure 2:**
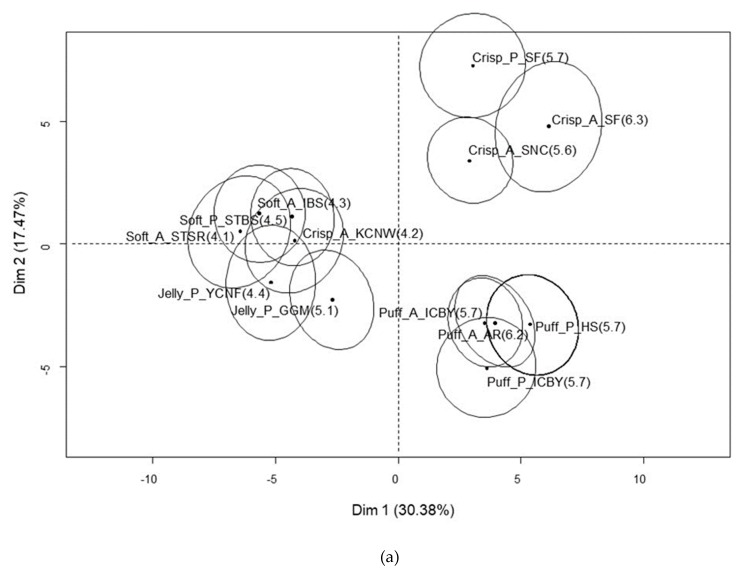

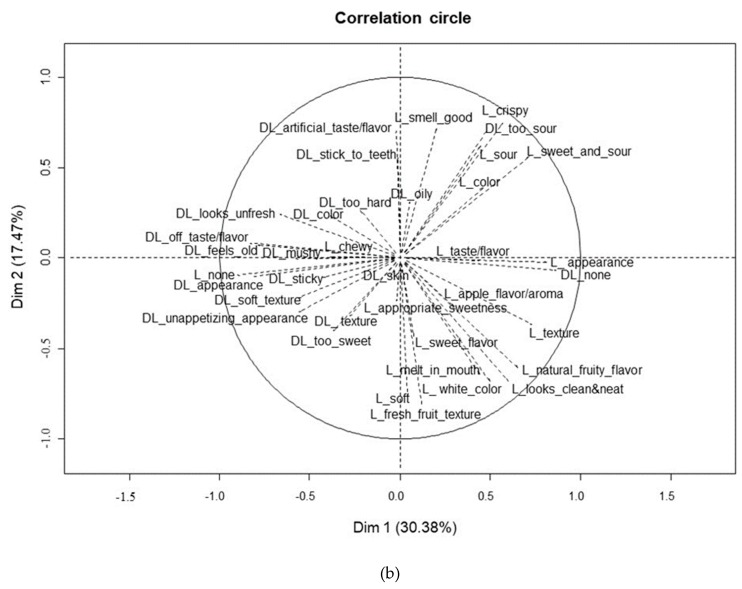
MFA plot of 13 dried fruit chips (**a**) and the reasons for liking and disliking them (**b**) among Chinese consumers.

A total of 47.9% variance was explained by Factor 1 (30.4%) and Factor 2 (17.5%) for Chinese consumers (Figure 2). F1 was mainly defined by the degree of liking of the samples. Thus, most of the crispy and puffy samples with OL > 5.5 were located on the positive F1 axis, while most of the soft and jelly-like samples as well as sample Crisp_A_KCNW were positioned on the negative F1. The crispy and puffy samples on the positive F1 axis showed strong positive correlations with liking the attributes of appearance and texture and no disliking. The jelly-like and soft samples showed strong positive correlations with off taste, looks unfresh, no liking, unappetizing, and generally disliking the appearance. F2 mainly indicated the contrast between the crispy samples on the positive axis and the puffy samples on the negative axis. Crispy samples with relatively high acceptance scores were characterized by liking reasons of crispiness, sourness, and sweetness/sourness, and disliking reasons of too sour, artificial taste, and sticking to the teeth. Puffy samples showed strong positive correlations with the liking reasons of soft, fresh fruit texture, clean appearance, natural fruity flavor, and melting in the mouth.

**Figure 3:**
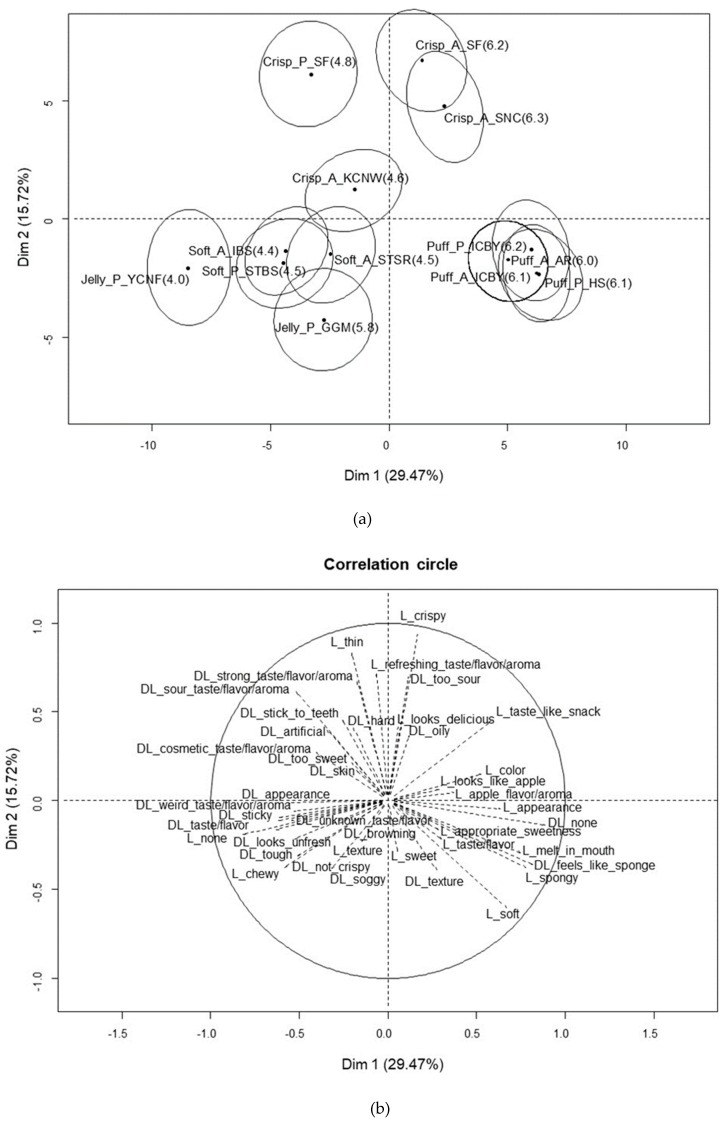
MFA plot of 13 dried fruit chips (**a**) and the reasons for liking and disliking them (**b**) among Korean consumers.

The MFA for Korean consumers showed that 45.2% of the variance was explained by F1 (29.5%) and F2 (15.7%) (Figure 3). Similar to the results for Chinese consumers, F1 was mainly defined by the degree of liking of the samples. The samples located on the positive F1 axis were characterized by liking attributes of appearance and appropriate sweetness and no disliking reasons, while those on the negative F1 axis were characterized by strange flavor, sticky, disliking the appearance/taste, and no liking reasons. The F2 axis was mainly defined by the level of crispiness present in the sample. Most of the crispy samples were positioned on the positive F2 axis, and the jelly-like and puffy samples were mainly on the negative F2 axis. The crispy samples were liked for their crispiness, thin size, and fresh taste, but disliked for their sour taste. Puffy samples located on the positive F1 and positive F2 axes were specifically described as melting in the mouth and soft as reasons for liking, while their sponginess was both liked and disliked. The soft and jelly-like samples located on the negative F1 and negative F2 axes were delineated by their stickiness, looking unfresh, toughness, not crisp, soggy, and browning as reasons for disliking.

**Figure 4:**
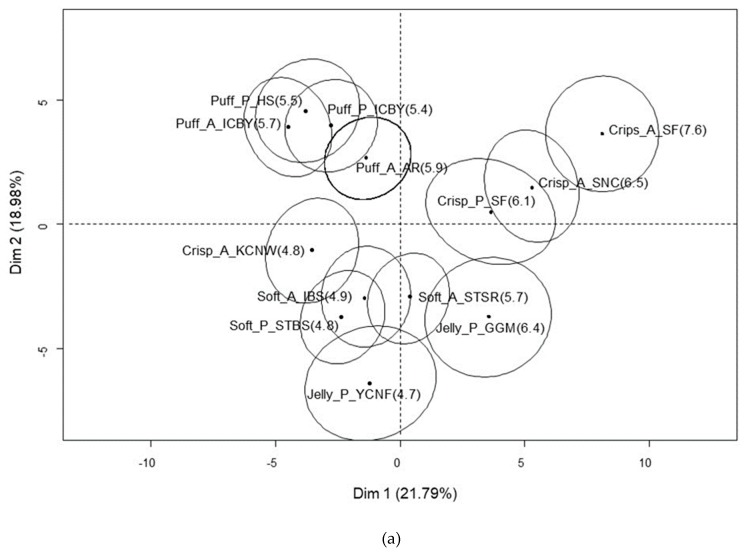

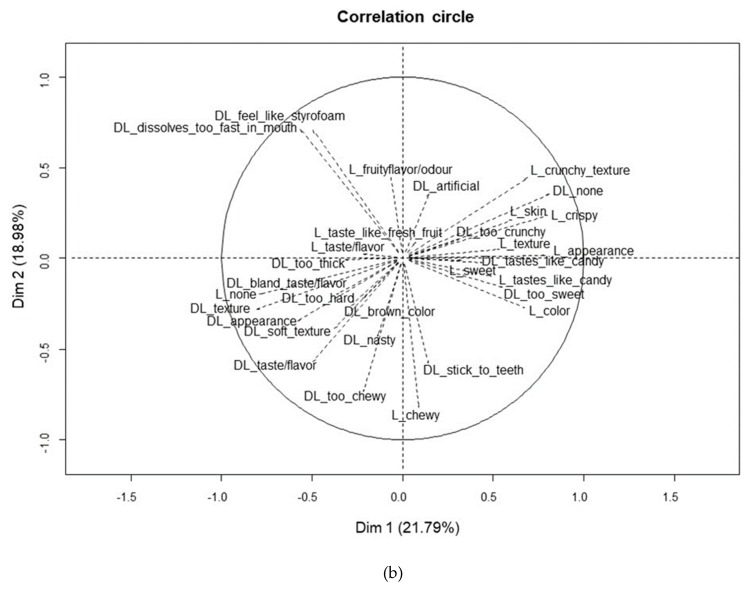
MFA plot of 13 dried fruit chips (**a**) and the reasons for liking and disliking them (**b**) among US consumers.

A total of 40.8% of the variance was explained by F1 (21.8%) and F2 (19.0%) in the MFA of US consumers (Figure 4). Similar to the consumers in the other two countries, the samples that the US consumers scored higher than 6.0 points for overall liking were positioned on the positive F1 axis, while other samples tended to be loaded on the negative F2 axis. These well-liked samples were commonly characterized by liking attributes of sweetness and appearance. The crispy samples were additionally liked for their crispy and crunchiness, whereas sample Jelly_P_GGM was liked for its color but disliked for tasting too sweet. F2 was mainly defined by the presence of a chewy texture. The soft and jelly-like samples were located on the negative F2 axis and showed a strong positive correlation with chewiness, which was both liked and disliked. Sticking to teeth and general taste/flavor were additional reasons for disliking the samples. Crispy and puffy samples were positioned on the positive F2 axis. Puffy samples were specifically characterized by the disliking attributes of dissolving too fast in the mouth and tasting like Styrofoam.

### 3.3. Hedonic Transfer of Texture Preference

The second aim of the study was to determine whether the preference for certain textures would transfer across different product categories. The individual texture preferences of consumers within the snack category were identified and their significance on delineating the fruit-chip texture acceptance was investigated using GLM analysis. Based on the snack texture preference, both Chinese and Korean consumers were grouped into likers of crispy, jelly-like, and puffy textures. The numbers of consumers who preferred crispy, puffy, and jelly-like snacks were similar in China and Korea. More than half of the consumers were classified as liking a crispy texture (27 Chinese and 32 Korean consumers), while smaller numbers of consumers were grouped into likers of a puffy texture (14 and 15, respectively) followed by likers of a jelly-like texture (12 and 9, respectively).

**Figure 5:**
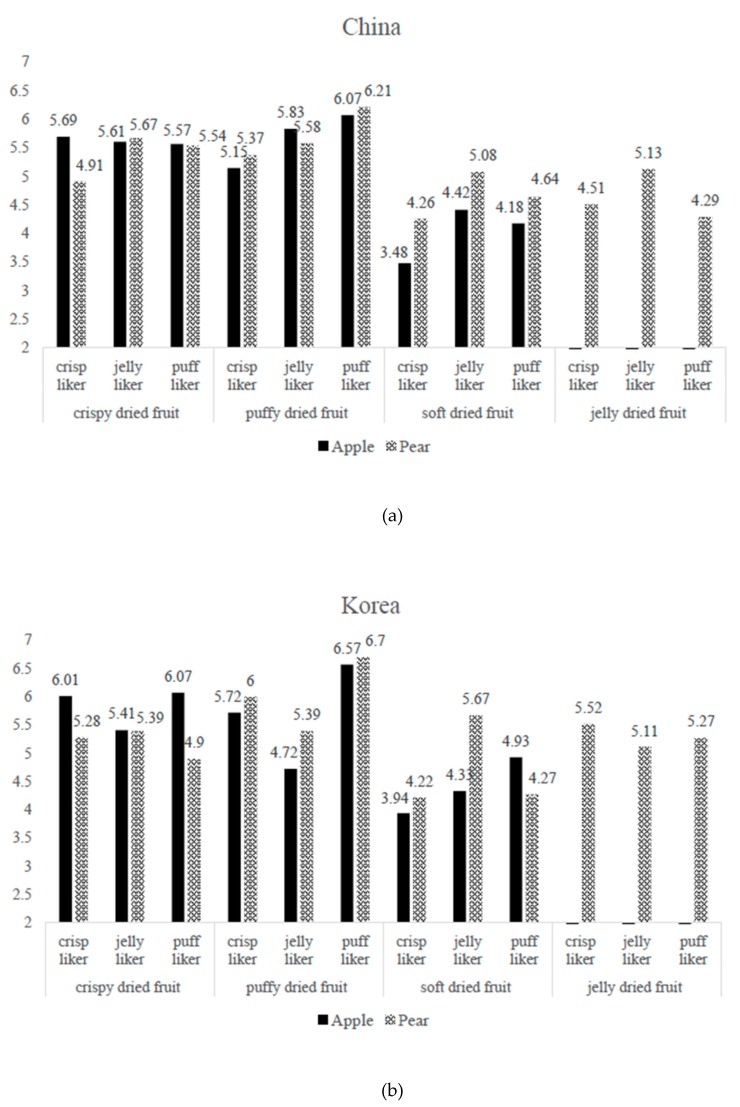
Effects of interaction between snack texture preferences and dried fruit-chip texture on the texture liking of dried apple and pear among Chinese (**a**) and Korean (**b**).

The results obtained when the GLM was applied separately to apple and pear chips showed that the OLs and texture liking (TL) scores were significantly affected by the interaction effect of snack texture preference and fruit texture type for both sample types (Table 6). The relationship between snack texture preference and the liking of apple and pear chips of various textures among Chinese and Korean consumers are shown in Figure 5a,b.

In the case of Chinese consumers, likers of a crispy texture scored the texture of crispy dried apple chips higher (TL = 5.7) than did the likers of puffy (TL = 5.2) and jelly-like (TL = 3.5) textures. Likers of a puffy texture scored the puffy dried fruit chips the highest for both apple and pear chips. Likers of a jelly-like texture gave a higher liking score than the other two groups for soft apple and pear dried fruit, although soft chips were still the least-preferred samples. For dried pear chips, Chinese likers of a crispy texture generally gave lower texture liking scores than likers of a jelly-like or puffy texture. In contrast, likers of a jelly-like texture tended to give higher scores for jelly-like fruit or compared to the other consumer groups.

The fruit-chip texture liking tendencies were slightly different for Korean consumers (Figure 5b). These likers of a jelly-like texture gave lower texture liking scores for crispy apple chips, puffy apple and pear chips compared to the other two groups, while likers of a puffy texture gave the highest score to all puffy fruit chips and a higher score to soft dried apple than did the other two groups. The texture likings of crispy or jelly-like dried pear were not strongly affected by their texture preferences, while likers of a jelly-like texture gave the highest score to soft dried pear.

## 4. Discussion

Many studies have investigated the drivers of food liking within a cross-cultural context. However, most of these studies have focused on flavor rather than texture aspects of foodstuff when investigating their acceptance, partly due to the nature of the product (e.g., beverages, sauces, and yogurts) but also due to the critical role that flavor quality plays in determining the acceptance level of a foodstuff [26,39,40,41,42]. Only a few studies have focused on the cross-cultural acceptance of foods with various texture properties [27,43]. The present study specifically performed experiments designed to understand the effect of texture characteristics on the perceptions of and preferences for fruit chips in a cross-cultural context. Although the flavors of the chips were intentionally varied by testing both apple and pear fruit chips together, flavor characteristics seemed to play a smaller role than texture since the variation in the acceptance score was smaller between apple and pear chips than among the different texture types.

We observed noticeable cross-cultural differences between Asian (Chinese and Korean) and US consumers in terms of fruit-chip liking. US consumers tended to give higher mean liking scores than Chinese and Korean consumers, which might be due to US consumers consuming fruit chips more frequently. Concerning the preferences for specific textures, general agreements were observed in the preference patterns for crispy and soft samples, with most consumers from all three countries giving higher acceptance scores to chips with crispy textures and lower scores to chips with soft textures.

In contrast, cross-cultural differences were found for the liking of puffy samples. The Chinese and Korean consumers liked puffy fruit chips as much as crispy ones, while US consumers gave significantly lower liking scores to puffy than crispy samples. When MFA was applied to the hedonic-based projective mapping with UFP, Chinese, Korean, and US consumers all described that the crispiness was the reason for liking crispy samples. This universal liking of a crispy texture is consistent with previous studies identifying this as one of the most important texture characteristics in snacks and fresh fruits [18,44,45]. Their appearance and the characteristic of melting in the mouth were the main drivers of liking puffy samples among Chinese and Korean consumers. However, US consumers disliked puffy samples because they considered that the samples dissolved too fast in the mouth and tasted like Styrofoam.

While familiarity per se was not frequently mentioned as a driver of liking or disliking in UFP, the consumers tended to find fruit chips more acceptable if the texture was familiar to them in the category of fruit chips or in terms of a broader snack category. That is, not only the familiarity with the fruit-chip texture itself, but also the contextual familiarity of the fruit-chip texture with that of the snacks available in the local markets of China, Korea, and the US seemed to play an important role in determining the cross-cultural differences in the liking of certain fruit-chip textures. Similar results were found in a cross-cultural acceptance study of yackwa, a traditional Korean sweet cookie [27]. Cross-cultural disagreements were shown for the types of texture preferred by Japanese, Korean, and French consumers. Consumers with different cultural backgrounds tend to like samples that have characteristics similar to those of products that they have consumed previously. As mentioned above, the US consumers in the present study generally consumed more fruit chips than did the Asian consumers. Moreover, the snacks with the largest market share in the US are chips (e.g., potato and tortilla), while extruded snacks have the largest share in the Chinese and Korean markets [46,47,48]. Chinese and Korean consumers are therefore more likely to be frequently exposed to extruded snacks eliciting puffy texture than are US consumers. Thus, consumers may have used the frame of reference constructed for fruit chips if they were frequent consumers of fruit chips, but also used the frame of reference for the broader snack category to evaluate their liking of fruit chips. However, this specific hypothesis remains to be validated in the future.

One of the similarities among the three consumer groups in this study is that they all disliked the soft dried fruits, using similar descriptions of being too chewy, soggy, and soft. These lower scores for soft dried fruits might be influenced by the frame of reference of the consumers for the fruit chips, which were crispy and crunchy. Josh et al. (2011) reported that consumers generally favored crispy-fried apple snack over less crispy one [49]. Similarly, in an investigation of the relationship between oral breakdown patterns and the preferences for biscuits, the 50 panelists demonstrated a consistent dislike of the softened samples, which could be seen from the results of the preference map of the biscuits [50]. Additionally, not all of the crisp samples were liked, and the consumers from all three countries described sample Crisp_A_KCNW being too hard as the reason for disliking it despite them liking crispy samples. The appropriateness of certain texture intensity was also the main criterion used by the consumers in the evaluations. Similarly, Jaworska and Hoffmann [51] found that the level of consumer acceptance of potato chips was only affected by the crispy texture intensity of the chips.

Previous studies have attempted to use the liking status for a certain flavor (e.g., sweetness or bitterness) as a predictor for the liking of food in another category that has similar sensory qualities. For example, Kim et al. [26] classified consumers based on their liking of sweet taste (as measured using sucrose solutions) and investigated the correlation with sweetness liking for orange juice as well as with the liking behavior in other food categories. Harwood et al. [52,53] classified consumers based on the self-declared liking status for milk chocolate versus dark chocolate, and used this to identify the rejection level of bitterness in chocolate-flavored products. These studies successfully demonstrated hedonic transfer from one product category to another; that is, significant differences in the acceptance level for different sweetness or bitterness of samples were observed among consumers with different sweetness or bitterness liking statuses, respectively. The present study also observed hedonic transfer from snack textures to fruit-chip textures. The presence of different texture liking statuses significantly affected the liking of consumers for the fruit-chip texture. For example, consumers who liked a puffy texture in a snack also gave higher liking scores for puffy fruit-chip samples.

While some successful cases of hedonic transfer have been described above, the liking status for specific attributes does not always effectively delineate how consumers will behave toward similar attributes. Several studies have attempted to use the sweet liking status to predict the consumption frequencies of sweet foods among children as well as adults [30,54]. Overall, the link between the sweet liking status and sweet food consumption has varied depending on the food category examined. Clearer hedonic transfer from one product category to another was observed if the product categories shared similar sensory characteristics or similar contextual product usage (e.g., from a water solution to orange juice, from solid chocolate to chocolate ice cream, or from snacks to fruit chips) [26,48]. Further investigations are necessary before drawing reliable conclusions about the food product categories in which hedonic transfer occurs.

Recently, Jeltima et al. (2015) classified consumers into four groups based on their oral behavior (cruchers, chewers, smooshers, and suckers) and proposed that these differences in oral behavior may influence the preference for food texture [55]. Our study has classified consumers into likers of crispy, jelly-like, or puffy texture, and shown that the liking of fruit chips was significantly affected by the consumer’s texture liking status of snacks. Investigating the oral behavior of the likers of crispy, jelly-like, or puffy texture may provide another way of understanding the hedonic transfer of textures in different product categories.

The present study carries several limitations to generalize the findings to a wider population due to a relatively small number of consumer participants from each country, a narrow age range of these participants (mostly in their 20s), and an imbalanced female to male ratio. Conducting a hedonic transfer experiment with only Asian consumers is another weakness of the study. Additionally, a lack of physico-chemical measurements (i.e., aroma compounds, free sugar content, total acidity, texture profile analysis, etc.) of the samples confines the interpretation of our findings on the drivers of (dis)liking for fruit chips from the practical perspective of product developers.

## 5. Conclusions

This study was designed to cross-culturally compare the acceptance and perceptual configuration of dried fruit chips among Chinese, Korean, and US consumers with the aim of predicting the potential market in these countries using hedonic-based projective mapping. The possibility of hedonic transfer concerning texture preferences across different product categories was also investigated. The key drivers of the liking of dried fruit chips was their crispness among the consumers from all three countries, with soft dried fruits being the least preferred dried fruits. Cross-cultural differences were observed in the acceptance of puffy fruit chips, with Chinese and Korean consumers liking puffy chips significantly more than did US consumers. These differences were mainly attributed to different degrees of familiarity with the texture in the context of fruit chips or snacks. Hedonic texture transfer from snacks to fruit chips was observed, with individual snack texture preferences significantly affecting the fruit-chip texture preferences of the consumers.

## Figures and Tables

**Table 1:** Product information for the dried fruit chips.

Fruit Type	Product Name	Origin	Manufacturer
Pear	Northwest pear chips (Crisp_P_SF)	USA	Sisters Fruit Company, Cornelius, OR, USA.
	Seneca pear chips (Crisp_P_SNC)	USA	Seneca Foods Corp., New York, NY, USA.
	Ichibiya freeze-dried pear chips (Puff_P_ICBY)	South Korea	Dami Dry Food Industry. Mungyeong, South Korea.
	Heswim pear chips (Puff_P_HS)	South Korea	Good Food Co., Ltd. Naju, South Korea.
	Starbucks real fruits pear (Soft_P_STBS)	South Korea	Midm Agricultural Union Co., Pyeongtaek, South Korea.
	GugGu miao li gan tiao(Jelly_P_GGM)	China	Ji ning shi gu gu miao Food Company, Jining, China.
	Yi cun nong fu (Jelly_P_YCNF)	China	Qing zhou shi ting you fu Food Company, Qingzhou, China.
Apple	Northwest apple chips (Crisp_A_SF)	USA	Sisters Fruit Company, Cornelius, Oregon, USA.
	Seneca apple chips (Crisp_A_SF)	USA	Seneca Foods Co. New York, NY, USA.
	Ichibiya freeze-dried apple Chips (Puff_A_ICBY)	South Korea	Dami Dry Food Industry. Mungyeong, South Korea.
	Apple ring (Puff_A_AR)	South Korea	Shingi Farm, Mungyeong, South Korea.
	Ibisak apple chips (Soft_A_IBS)	South Korea	Hephzibah Food and Beverage, Naju, South Korea.
	Sol to sa rang apple chips (Soft_A_STSR)	South Korea	Daon Food, Seoul, South Korea.
	Kyeong chang nong won apple chips (Crisp_A_KCNW)	South Korea	Safe Food, Busan, South Korea.

**Table 2:** Product information for the snacks.

Product Name	Origin	Manufacturer
Honey butter maple syrup chips (Crisp_MS)	South Korea	Haitai Confectionery & Foods Co., Seoul, South Korea
Terra sweet potato chips (Crisp_SP2)	USA	The Hain Celestial Group, Inc, New York, NY, USA
Coconut chips (Crisp_C)	Thailand	Thitinan Bee Food Co., Bangkok, Thailand
Korea sweet potato chips (Crisp_SP1)	South Korea	Lotte Confectionery Co. Seoul, South Korea ood Food Co., South Korea
Apple and yogurt cubes (Puff_A)	South Korea	GoodFood Co. Naju, South Korea.
Handmade meringue-white, non-artificial pigment (Puff_M2)	South Korea	Miacake, Busan, South Korea
Aramonde meringue (Puff_M1)	South Korea	Aramonde, Seoul, South Korea
Fruit by the foot jelly -grape and green grape flavor (Jelly_G)	USA	General Mills, Minneapolis, MN, USA
Juicy mango soft jelly (Jelly_MG)	China	Fuijia Foods Co., Quanzhou, China

**Table 3:** *F*- and *p*- values associated with statistical significance for 13 types of dried fruit.

Factor	Attributes	*F*-Value	*p*-Value
Sample	Overall liking	33.092	0.000
	Appearance liking	51.220	0.000
	Taste and flavor liking	18.295	0.000
	Texture liking	40.588	0.000
Nationality	Overall liking	5.131	0.007
	Appearance liking	3.755	0.025
	Taste and flavor liking	7.817	0.001
	Texture liking	1.358	0.260
Nationality × Sample	Overall liking	3.596	0.000
	Appearance liking	8.340	0.000
	Taste and flavor liking	2.881	0.000
	Texture liking	3.318	0.000
Nationality × Panelist	Overall liking	3.193	0.000
	Appearance liking	3.189	0.000
	Taste and flavor liking	3.141	0.000
	Texture liking	3.634	0.000

× refers to interaction effect of the independent factors

**Table 4:** Mean ± standard deviation of sample liking scores evaluated by Chinese, Korean, and US consumers.

Attributes		Overall Liking			Appearance Liking			Taste and Flavor Liking			Texture Liking		
	**Nationality**	CHN	KOR	USA	CHN	KOR	USA	CHN	KOR	USA	CHN	KOR	USA
**Sample**													
Crisp_A_KCNW		4.2 ± 1.4 ^a*^	4.6 ± 2.0 ^ab^	4.8 ± 1.9 ^ab^	3.8 ± 1.3 ^a^	3.7 ± 1.3 ^a^	4.7 ± 1.8 ^ab^	4.6 ± 1.5 ^a^	5.2 ± 2.0 ^cd^	5.7 ± 2.1 ^abc^	4.4 ± 1.8 ^ab^	4.7 ± 2.2 ^abc^	4.4 ± 2.2 ^ab^
Crisp_A_SF		6.3 ± 1.8 ^f^	6.2 ± 1.8 ^c^	7.6 ± 1.4 ^f^	6.4 ± 1.6 ^e^	6.5 ± 1.5 ^g^	7.5 ± 1.4 ^g^	6.1 ± 1.9 ^de^	5.7 ± 2.0 ^defg^	7.5 ± 1.7 ^e^	6.6 ± 1.8 ^f^	6.9 ± 1.4 ^g^	7.4 ± 1.5 ^f^
Crisp_A_SNC		5.6 ± 1.7 ^de^	6.3 ± 1.7 ^c^	6.5 ± 1.8 ^e^	5.7 ± 1.6 ^d^	5.4 ± 1.6 ^de^	6.8 ± 1.9 ^f^	5.6 ± 1.7 ^bcd^	6.3 ± 1.6 ^g^	6.4 ± 1.9 ^cd^	5.9 ± 1.5 ^e^	6.2 ± 1.8 ^fg^	6.8 ± 1.8 ^ef^
Puff_A_AR		6.2 ± 1.5 ^ef^	6.0 ± 2.0 ^c^	5.9 ± 1.9 ^cde^	6.5 ± 1.5 ^e^	6.6 ± 1.4 ^g^	6.6 ± 1.6 ^ef^	6.4 ± 1.6 ^e^	6.4 ± 1.6 ^g^	6.2 ± 1.8 ^cd^	5.6 ± 2.2 ^de^	5.4 ± 2.3 ^de^	5.2 ± 2.3 ^bc^
Puff_A_ICBY		5.6 ± 1.7 ^de^	6.1 ± 2.0 ^c^	5.7 ± 2.0 ^cd^	6 ± 1.4 ^de^	6.3 ± 1.7 ^fg^	5.3 ± 2.1 ^bc^	5.8 ± 1.5 ^cde^	6.0 ± 1.9 ^efg^	6.3 ± 1.9 ^cd^	5.5 ± 1.7 ^de^	6.0 ± 2.1 ^ef^	5.3 ± 2.2 ^cd^
Soft_A_IBS		4.3 ± 1.6 ^ab^	4.4 ± 1.8 ^ab^	4.9 ± 1.7 ^ab^	4.8 ± 1.3 ^c^	4.8 ± 1.4 ^bc^	5.9 ± 1.7 ^cd^	4.8 ± 1.7 ^a^	4.7 ± 1.9 ^bc^	5.1 ± 2.1 ^ab^	3.8 ± 1.9 ^a^	4.1 ± 1.8 ^a^	4.1 ± 1.7 ^a^
Soft_A_STSR		4.1 ± 1.5 ^a^	4.5 ± 1.9 ^ab^	5.7 ± 1.8 ^cd^	4.1 ± 1.3 ^a^	4.6 ± 1.6 ^bc^	6.0 ± 1.7 ^de^	4.8 ± 1.7 ^ab^	5.4 ± 1.7 ^de^	6.1 ± 1.9 ^cd^	3.9 ± 1.6 ^a^	4.2 ± 1.9 ^a^	5.1 ± 2.4 ^bc^
Crisp_P_SF		5.7 ± 2.1 ^de^	4.8 ± 2.0 ^b^	6.1 ± 2.2 ^cde^	6.0 ± 1.6 ^de^	5.6 ± 1.7 ^de^	7.0 ± 1.4 ^fg^	5.8 ± 2.3 ^cde^	5.2 ± 2.1 ^cd^	5.9 ± 2.5 ^c^	5.7 ± 2.0 ^de^	5.1 ± 2.1 ^cd^	6.0 ± 2.1 ^de^
Jelly_P_GGM		5.1 ± 1.9 ^cd^	5.8 ± 2.0 ^c^	6.4 ± 2.0 ^de^	5.0 ± 1.9 ^c^	5.4 ± 1.8 ^de^	5.6 ± 2.1 ^cd^	5.2 ± 1.9 ^abc^	5.8 ± 1.9 ^defg^	6.8 ± 1.9 ^d^	5.1 ± 1.9 ^bcd^	5.9 ± 1.8 ^ef^	6.4 ± 2.1 ^e^
Jelly_P_YCNF		4.4 ± 2.1 ^ab^	4.0 ± 1.7 ^a^	4.7 ± 2.3 ^a^	4.6 ± 1.8 ^bc^	4.4 ± 1.5 ^b^	4.9 ± 1.9 ^ab^	4.9 ± 1.9 ^a^	4.1 ± 1.9 ^a^	4.9 ± 2.2 ^a^	4.1 ± 1.9 ^a^	4.9 ± 1.9 ^bcd^	4.9 ± 2.4 ^bc^
Puff_P_HS		5.7 ± 1.5 ^de^	6.1 ± 2.0 ^c^	5.5 ± 2.0 ^bc^	5.7 ± 1.6 ^d^	5.9 ± 2.0 ^ef^	4.4 ± 2.0 ^a^	5.9 ± 1.3 ^de^	5.9 ± 1.9 ^efg^	6.1 ± 2.0 ^cd^	5.8 ± 1.6 ^de^	6.3 ± 2.0 ^fg^	5.1 ± 2.4 ^bc^
Puff_P_ICBY		5.7 ± 1.6 ^de^	6.2 ± 2.0 ^c^	5.4 ± 2.2 ^abc^	5.7 ± 1.4 ^d^	5.9 ± 1.6 ^ef^	4.6 ± 2.1 ^ab^	5.8 ± 1.5 ^cde^	6.1 ± 1.6 ^fg^	5.8 ± 2.2 ^bc^	5.7 ± 1.6 ^de^	5.9 ± 2.1 ^ef^	5.1 ± 2.4 ^bc^
Soft_P_STBS		4.5 ± 1.6 ^abc^	4.5 ± 1.7 ^ab^	4.8 ± 1.9 ^a^	4.2 ± 1.4 ^ab^	4.3 ± 1.4 ^b^	4.3 ± 1.9 ^a^	5.1 ± 1.7 ^a^	5.4 ± 1.6 ^def^	5.7 ± 1.8 ^abc^	4.5 ± 1.8 ^ab^	4.4 ± 1.9n ^ab^	3.9 ± 2.1 ^a^

^*^ Means not sharing a common lowercase letter within the same column are significantly different among samples at *p* < 0.05.

**Table 5:** Liking and disliking terms selected by more than 10% of the Chinese, Korean, and US consumers.

CHN		KOR		USA	
Liking	Disliking	Liking	Disliking	Liking	Disliking
appearance apple flavor/aroma appropriate sweetness chewy color crispy fresh fruit texture looks clean & neat melt in mouth natural fruity flavor none smell good soft sour sweet and sour sweet flavor taste/flavor texture white color	appearance artificial taste/flavor color feels old looks unfresh mushy none off taste/flavor oily skin soft texture stick to teeth sticky texture too hard too sour too sweet unappetizing appearance	appearance apple flavor/aroma appropriate sweetness chewy color crispy looks delicious looks like apple melt in mouth none refreshing taste/flavor/odor soft spongy sweet taste/flavor taste like snack texture thin	appearance artificial browning cosmetic taste/flavor/aroma feels like sponge hard looks unfresh none not crispy off taste/flavor/aroma oily skin soggy sour taste/flavor/aroma stick to teeth sticky strong taste/flavor/aroma taste/flavor texture too sour too sweet tough unknown taste/flavor	appearance chewy color crispy crunchy texture fruity flavor/aroma none skin sweet taste/flavor taste like fresh fruit tastes like candy texture	appearance artificial bland taste/flavor brown color dissolves too fast in mouth feel like Styrofoam nasty none soft texture stick to teeth taste/flavor tastes like candy texture too chewy too crunchy too hard too sweet too thick

**Table 6:** *F*- and *p*-values associated with statistical significance for seven types of dried apple and pear chips.

		Apple		Pear	
Factor	Attributes	*F*-Value	*p*-Value	*F*-Value	*p*-Value
Texture preference of snack	Overall liking	1.117	0.331	0.970	0.382
	Appearance liking	1.669	0.193	0.342	0.711
	Taste/flavor liking	0.208	0.813	1.848	0.162
	Texture liking	2.761	0.068	1.142	0.323
Dried fruit texture type	Overall liking	35.642	0.000	15.185	0.000
	Appearance liking	58.803	0.000	22.827	0.000
	Taste/flavor liking	17.929	0.000	9.990	0.000
	Texture liking	47.874	0.000	12.850	0.000
Nationality	Overall liking	0.513	0.475	0.188	0.666
	Appearance liking	0.160	0.690	0.341	0.561
	Taste/flavor liking	0.526	0.470	0.000	0.984
	Texture liking	0.732	0.394	0.993	0.321
Dried fruit texture type × Nationality	Overall liking	0.948	0.388	2.541	0.055
	Appearance liking	0.745	0.475	1.694	0.167
	Taste/flavor liking	0.857	0.425	2.009	0.111
	Texture liking	0.577	0.562	1.922	0.125
Texture preference of snack × Nationality	Overall liking	1.435	0.243	0.090	0.914
	Appearance liking	0.928	0.398	0.675	0.511
	Taste/flavor liking	1.528	0.222	0.842	0.434
	Texture liking	1.661	0.195	0.566	0.569
Texture preference of snack × Dried fruit texture type	Overall liking	3.773	0.005	2.393	0.027
	Appearance liking	1.371	0.242	4.107	0.000
	Taste/flavor liking	2.935	0.020	1.012	0.417
	Texture liking	3.425	**0.009**	2.731	**0.013**
Texture preference of snack × Dried fruit texture type × Nationality	Overall liking	0.634	0.638	0.393	0.883
	Appearance liking	1.443	0.218	0.469	0.831
	Taste/flavor liking	0.758	0.553	0.777	0.588
	Texture liking	0.612	0.654	0.857	0.526
Texture preference of snack × Nationality × Panelist	Overall liking	2.767	0.000	2.391	0.000
	Appearance liking	1.955	0.000	2.487	0.000
	Taste/flavor liking	2.606	0.000	2.085	0.000
	Texture liking	2.720	0.000	2.444	0.000

× refers to interaction effect of the independent factors.

## Appendix A

**Table A1:** *F*- and *p*- values associated with statistical significance for dried fruit texture types on the liking ratings of apple and pear chip samples.

Fruit Type	Factor	Attributes	*F*-Value	*p*-Value
Apple	Dried fruit texture type	Overall liking	57.759	0.000
		Appearance liking	42.182	0.000
		Taste and flavor liking	34.661	0.000
		Texture liking	87.569	0.000
	Nationality	Overall liking	5.965	0.003
		Appearance liking	12.009	0.000
		Taste and flavor liking	7.824	0.001
		Texture liking	1.451	0.237
	Dried fruit texture type ×^1^ Nationality	Overall liking	4.799	0.001
		Appearance liking	13.194	0.000
		Taste and flavor liking	2.623	0.033
		Texture liking	3.053	0.016
	Nationality ×^1^ Panelist	Overall liking	2.042	0.000
		Appearance liking	1.766	0.000
		Taste and flavor liking	2.058	0.000
		Texture liking	2.152	0.000
Pear	Dried fruit texture type	Overall liking	17.353	0.000
		Appearance liking	41.936	0.000
		Taste and flavor liking	8.850	0.000
		Texture liking	22.692	0.000
	Nationality	Overall liking	2.070	0.129
		Appearance liking	0.075	0.928
		Taste and flavor liking	3.100	0.047
		Texture liking	0.151	0.860
	Dried fruit texture type ×^1^ Nationality	Overall liking	5.156	0.000
		Appearance liking	12.378	0.000
		Taste and flavor liking	2.383	0.027
		Texture liking	7.124	0.000
	Nationality ×^1^ Panelist	Overall liking	1.804	0.000
		Appearance liking	1.915	0.000
		Taste and flavor liking	1.895	0.000
		Texture liking	1.922	0.000

×^1^ refers to interaction effect of the independent factors.
